# Method for generating transparent porcine tibia showing the intraosseous artery

**DOI:** 10.1186/s13018-022-03302-2

**Published:** 2022-09-05

**Authors:** Hongyu Wang, Jiaming Wan, Kailong Geng, Xiangnan Zhang, Ruixing Hou

**Affiliations:** 1 Suzhou Medical College of Soochow University, Suzhou, China; 2 Teaching Hospital of the Medical College of Yangzhou University, Suzhou Ruihua Orthopedic Hospital, Yangzhou, China; 3 Suzhou Ruihua Orthopedic Hospital, Suzhou, China

**Keywords:** 3D printing, Corrosion, Perfusion, Tibia

## Abstract

**Background:**

The occurrence of nonunion after tibial fracture surgery is mainly related to insufficient blood supply. Therefore, anatomical study of the internal and external tibial artery is very important, but there is no good method for displaying the intraosseous artery clearly and intuitively. This hinders the protection and reconstruction of it by surgeons, as well as the development of new instruments and techniques by researchers.

**Objective:**

To develop a transparent specimen that could clearly display the intraosseous artery of the tibia.

**Methods:**

In 10 isolated pig calves with popliteal vessels, the popliteal artery was exposed and a tube was placed. A casting agent was then injected at constant pressure, and the tissue around the blood vessel was preliminarily removed after solidification. The perivascular tissue and periosteum were further removed via alkali corrosion, and the tibia was fixed with an external fixator to protect the non-corrosive areas at both ends. Alternate acid corrosion and flushing were then applied until the intraosseous artery was completely exposed. The distribution and branches of intraosseous nutrient arteries were observed with the naked eye and via microscopy. Three-dimensional (3D) scanning and 3D printing filling techniques were used to make transparent tibia specimens with preservation of intraosseous arteries.

**Results:**

A cast specimen of the intraosseous artery of porcine tibia was successfully generated via epoxy resin perfusion combined with acid–alkali etching, and the intraosseous artery was clearly visible. The 3D printing and filling technique successfully produced a transparent tibia specimen with preservation of internal bone arteries, and accurately restored the external shape of the tibia. The foramen of the nutrient artery appeared near the middle upper third of the lateral edge of the tibia. After entering the tibia, the nutrient artery proceeded forward, medial, and downward for a certain distance, twisted and turned near the midpoint of the medullary cavity, and divided into the ascending and descending branches. After going in the opposite direction for a distance, the ascending trunk sent out 2–3 branches, and the descending trunk sent out 2–3 branches.

**Conclusion:**

The cast specimen of pig intraosseous artery generated via the above-described perfusion corrosion method provides methodological guidance for the study of anatomical characteristics of the intraosseous artery, and a theoretical basis for the study of new methods of internal fixation and reconstruction of the blood supply of the lower tibia.

## Background

Because of the particularity of the physiological structure of the leg, the tibia is more prone to open fracture and nonunion than other long bones [1]. The incidence of postoperative nonunion of a tibial fracture is currently 7%–12% [2]. The majority of such patients are unable to return to their pre-injury state of activity, their risk of thrombosis and pendant pneumonia increases, and they experience much longer recovery times and greater financial costs than those without nonunion. These outcomes place a huge burden on both the patients and society [3].

A large number of experimental results and clinical evidence show that the decrease in blood supply at the fracture end of a fractured tibia is one of the important factors leading to nonunion [4–7]. Tibial shaft fracture can easily damage the periosteum and intraosseous vessels. In fracture fixation, although re-injury of the remaining periosteal vessels can be avoided under direct vision, iatrogenic injury of intraosseous vessels is difficult to avoid due to the lack of an effective means of observation [8]. Therefore, a clear understanding of the physiological structure of the intraosseous artery will help to avoid damage to the intraosseous artery during internal fixation, and is of great significance for early blood supply reconstruction to prevent tibial nonunion.

The intraosseous vessels of the tibia are mainly derived from the nutrient artery. In the past few decades some scholars have summarized the origin and course of nutrient arteries outside the bone via anatomical or imaging studies of human specimens [9, 10]. However, there is no better method to clearly and intuitively display the nutrient arteries in the tibia, and there is also a lack of relevant visual models for use in clinical teaching. In the current study transparent porcine tibia specimens showing intraosseous arteries were generated via an acid–alkali corrosion and 3D scanning and printing process. It is expected to provide methodological guidance for further study of the anatomical characteristics of the intraosseous artery, and a theoretical basis for the study of internal fixation and reconstruction of the blood supply to the lower tibia.

## Materials and methods

### Materials

Ten isolated pig calves with popliteal vessels from ordinary Landrace pigs aged 8–12 months (mean 10 months) and weighing approximately 150 kg were used in the study. The study was approved by the Ethics Committee of Ruihua Hospital, affiliated with Soochow University, China (approval number RX2019003). Epoxy resins were purchased from the Dongguan Xiaoma New Material Technology Co., Ltd. (Cat No. 318AB-C2-1 kg). Kits contained solutions A and B. The main component of solution A was epoxy colloid ((C_11_H_12_O_3_)n), and the main component of solution B was polyetheramine curing agent (C_3_H_10_N_2_O). Other materials used in the study included a manometer (SanLiang, DP370), UV-curing adhesive (Kisling AG, ergo8500), a handheld 3D scanner (Nanjing Weibu 3D Technology Co., Ltd., REEYEEPRO +), a 3D printer (Guangzhou Maipu Regenerative Medical Technology Co., Ltd., SLJ-JUPU 420), No. 8, 3.0 Maximum Mold Release Wax (MIRACLE GLOSS, M0811V3), and surgical light microscope with 180° positions for use by two people with the same optical path, same magnification, and same orientation (Zhenjiang Zhuo Chuang Medical Technology Co., Ltd., ZC-X-4A).

### Vascular casting

The vascular casting medium was prepared by mixing epoxy A solution and epoxy B solution (3:1, m/m), followed by the addition of 4 drops of red metal complex dye [11]. The preparation was then mixed thoroughly, then rested for 30 min prior to use.

**Fig. 1:**
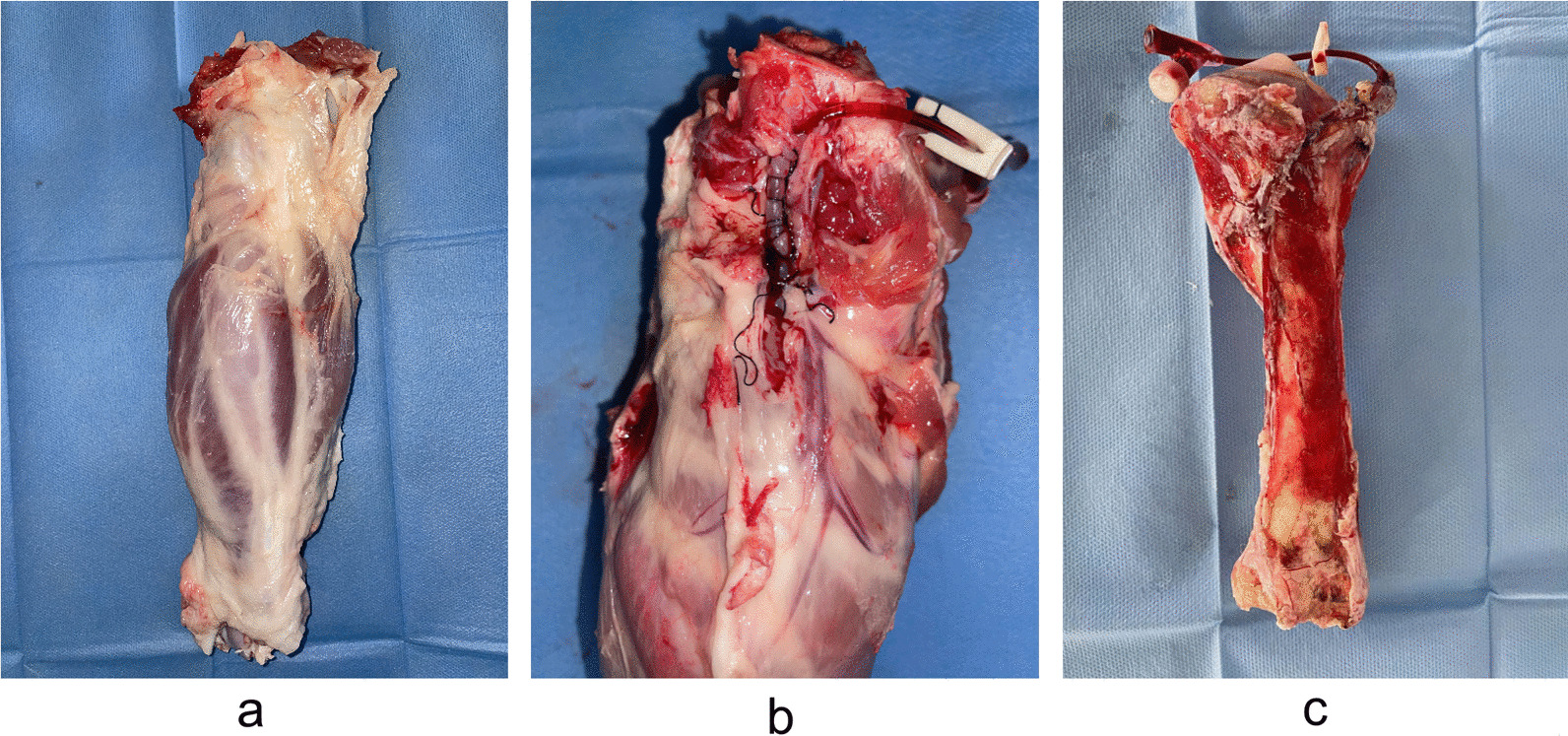
Perfusion and anatomy of the tibia. **a** Calf specimen. **b** Popliteal artery catheterization. **c** Preliminary removal of soft tissue

The skin was cut at the popliteal fossa, the muscle space was opened layer by layer, and the popliteal artery was located in front of the knee capsule. The popliteal artery was dissected from 6–8 cm by oblique dissection along the outer edge of the semitendinosus muscle. The indwelling needle was removed and inserted into the popliteal artery, which was ligated and fixed. The remaining blood was washed with 625 U/mL heparin saline until the distal arteriovenous effluent was clear and transparent, and the broken branches were ligated at that time [12]. The indwelling needle was connected to the manometer and the syringe, and the vascular casting agent was injected under a constant pressure of 25 kPa [13, 14]. After vascular filling, reperfusion was performed every 15 min to maintain the filled state of the popliteal artery. After perfusion the leg specimens were incubated at room temperature for 24 h, then dissected along the popliteal artery. The surrounding soft tissue was then removed, and the tibia and peripheral artery were preserved (Fig. 1).

**Fig. 2:**
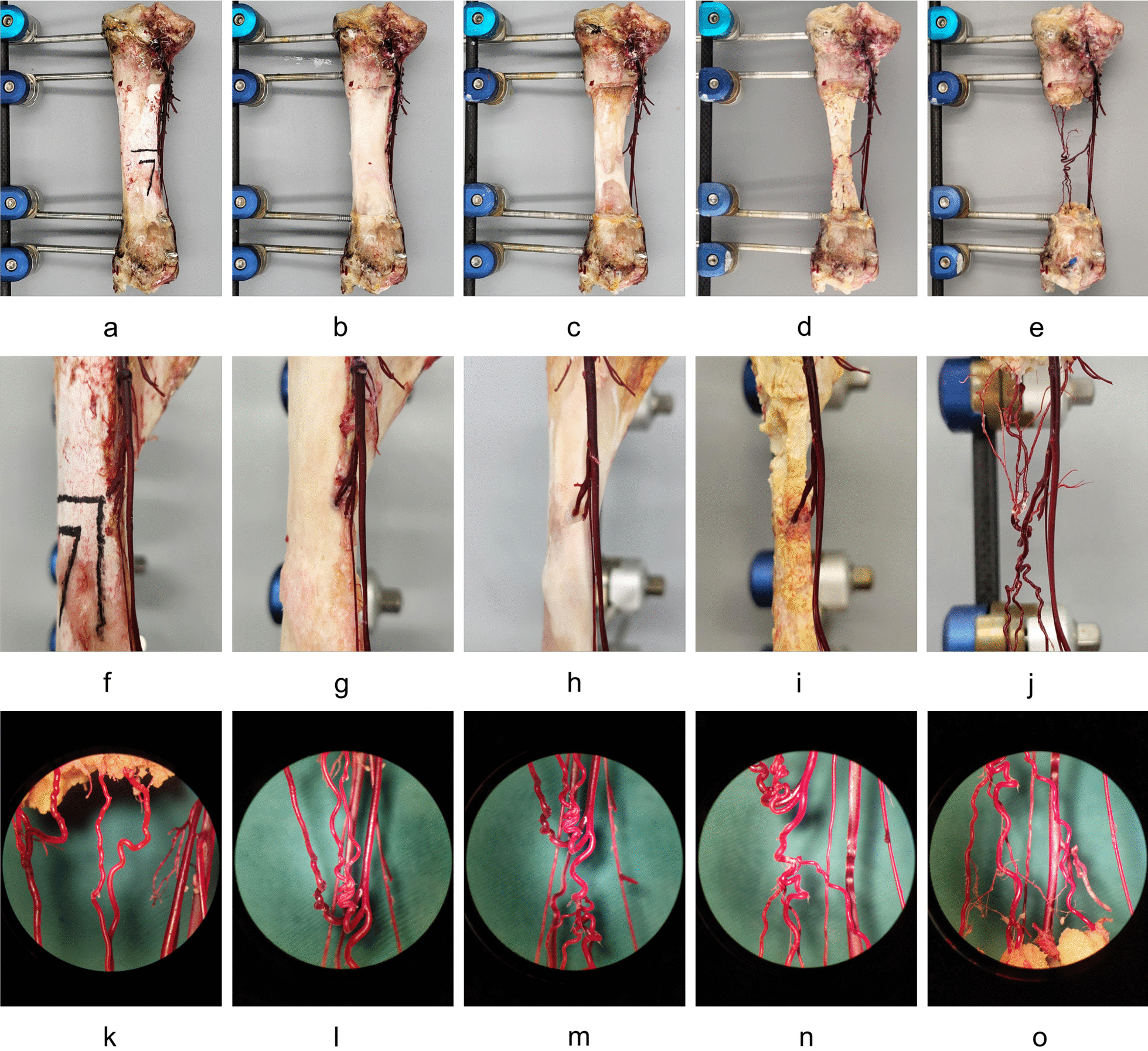
Corrosion process. **a** External fixation bracket. **b–e** 1st–4th acid corrosion. **f–j** Exposure of nutrient arteries. **k–o** Vascular branches viewed under microscope (10 ×)

A total of four 5.0-mm-diameter pins were implanted at 1.5 cm and 3.0 cm below the tibial plateau, and 1.5 cm and 3.0 cm above the ankle plane, with the anterior edge of the tibia as the landmark line. The pins were firmly connected with external fixators (Fig. 2a). After the tibia was completely dry, the upper and lower ends of the tibia and the bone pin were completely covered and sealed with UV curing adhesive to prevent the bone pin from loosening or breaking in the follow-up operation due to corrosion. The tibia was completely immersed in the flume containing 10% HCl solution, and at 12-h intervals impurities were removed under a microscope and the solution was replaced [15, 16], until the vascular casting was completely exposed (Fig. 2b–2j). The distribution and branches of bone nutrient arteries were recorded and statistically analyzed by the naked eye and via microscopy (10 ×) (Fig. 2k–2o).

### Transparent tibia generation

**Fig. 3:**
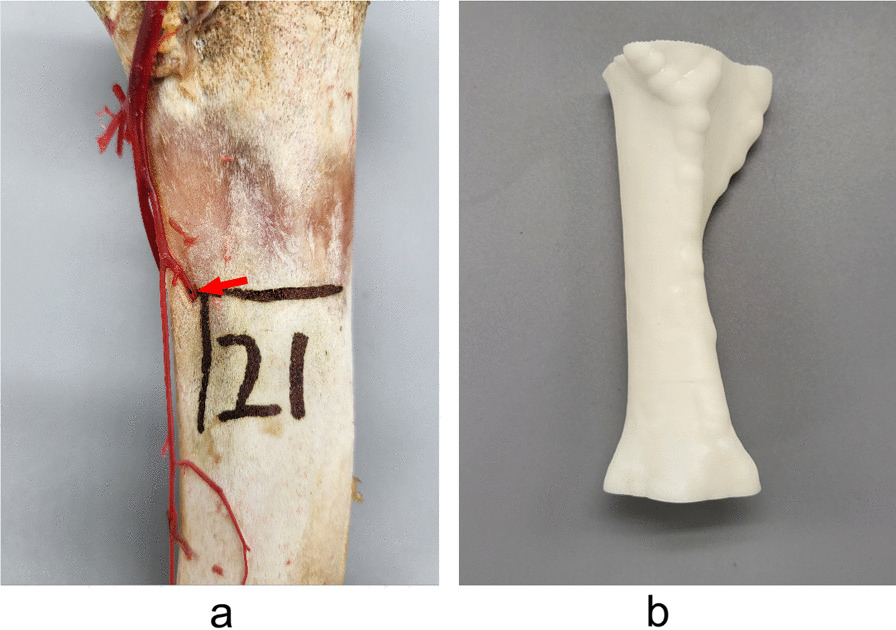
**a** The red arrow is the foramen of the nutrient artery. **b** Resin mold

The preliminarily treated tibia was completely immersed in 50% NaOH solution for alkali corrosion for 6 h, then it was washed with running water for 5 min, and the residual soft tissue on the surface of the tibia was completely removed [12]. The foramen of the nutrient artery on the tibia was located, and its location was marked (Fig. 3a). The tibia was stood upright on the scanning disk and modelled with a 3D scanner. Wiboox Reeyee-Plus was used to remove the excess and encapsulate it into a closed model. The middle part of the tibia was delineated in Materialise Magics 21.0 as the printing range, and the printing thickness was magnified by 1.15 × 2.00 mm to make a resin mold (Fig. 3b).

**Fig. 4:**
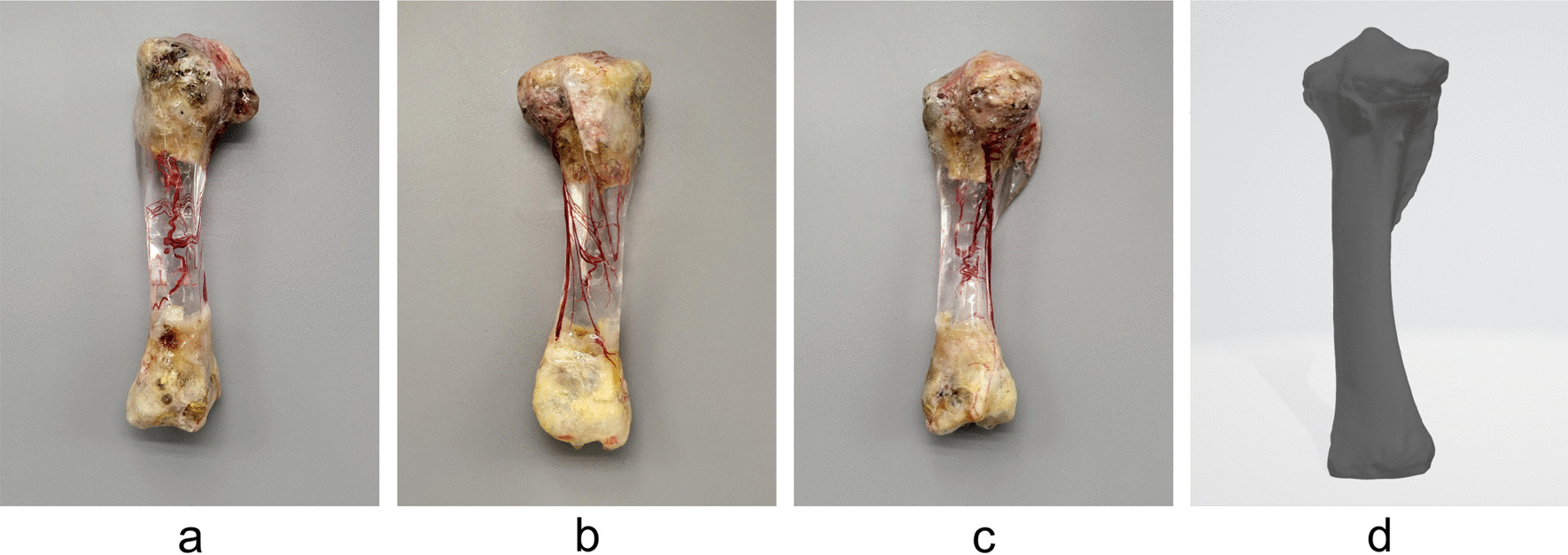
Transparent tibia. **a** Rear view. **b** Side view. **c** Front view. **d** 3D model

The position of the arterial nutrient foramen was marked via comparison of the resin mold with the 3D image. The mold was divided into three parts by the long axis of the tibia where the nutrient hole was located; the long axis of the anterior edge of the tibia and the long axis of the medial edge of the tibia. No. 8, 3.0 Maximum Mold Release Wax was evenly applied onto the inner surface of the mold, and the process was repeated three times. The mold was installed on the corroded tibia and fixed with glue, then tightness was tested by injecting water into the reserved perfusion port. After assessing the tightness, the newly configured epoxy resin liquid was injected from the perfusion port to be completely filled. The static time at room temperature was at least 72 h. The mold was removed after the epoxy resin was completely dry, irregular areas on the surface of the tibia were polished, and additional polishing was conducted until the red blood vessels inside the resin were clearly exposed (Fig. 4a–4c).

### Statistical analysis

SPSS 21.0 software (IBM SPSS Statistics for Windows, Jiangsu, Suzhou, China) was used for statistical analysis. Percentage (%) is used to describe the location of the nutrient hole. Other data are expressed as means ± the standard deviation.

## Results

### The cast specimen of the tibial artery is clear and complete

**Fig. 5:**
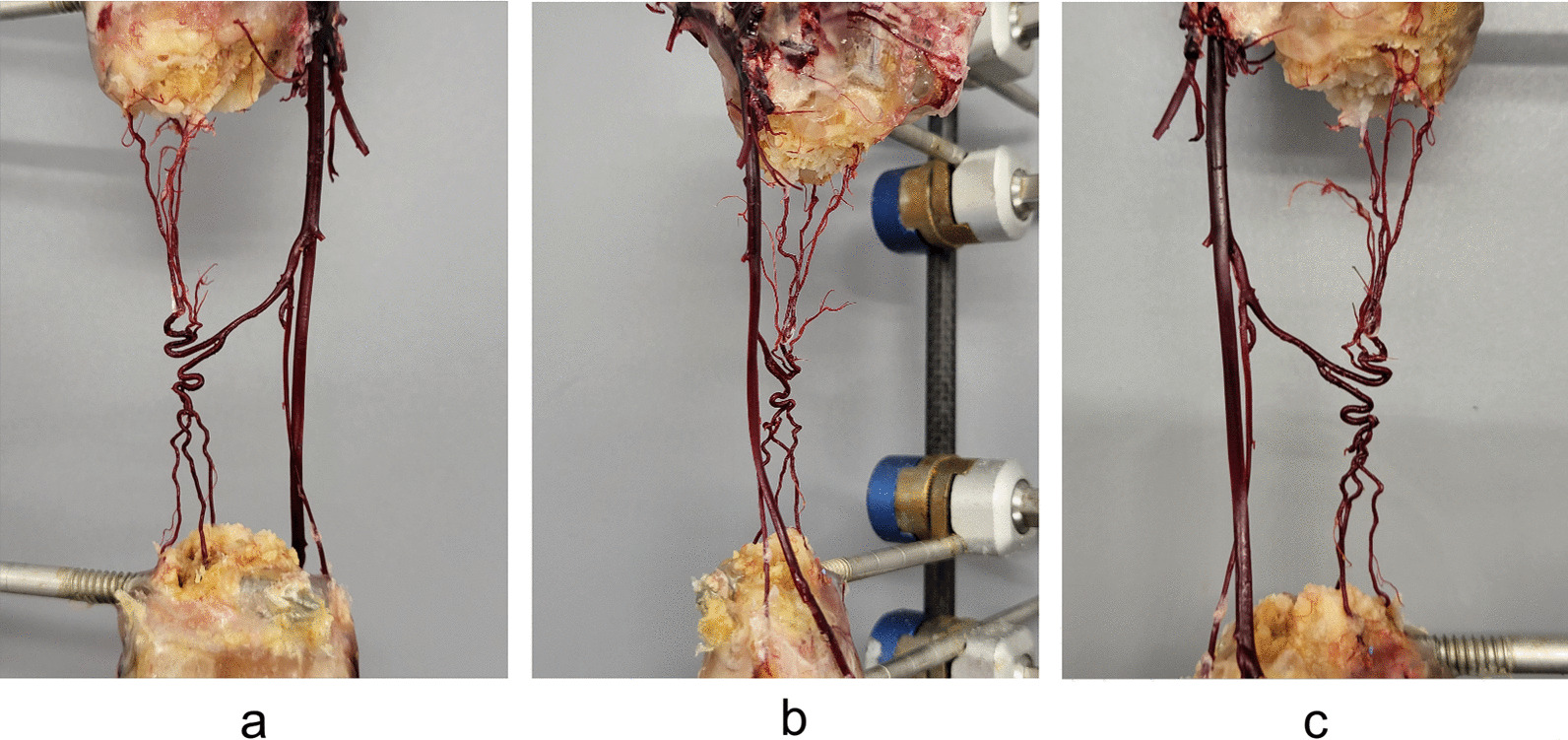
Vascular casting. **a** Rear view. **b** Side view. **c** Front view

After perfusion with epoxy resin, the complete vascular network of the internal tibial artery was evident (Fig. 5).

### The transparent tibia restored the original shape of the natural tibia

Combined with 3D printing technology, the original opaque bone can be replaced with a transparent medium that can reveal the internal vascular network, and the external shape of the tibia can be accurately restored (Fig. 4c–4d).

### Distribution of nutrient artery foramen and intraosseous artery

**Fig. 6:**
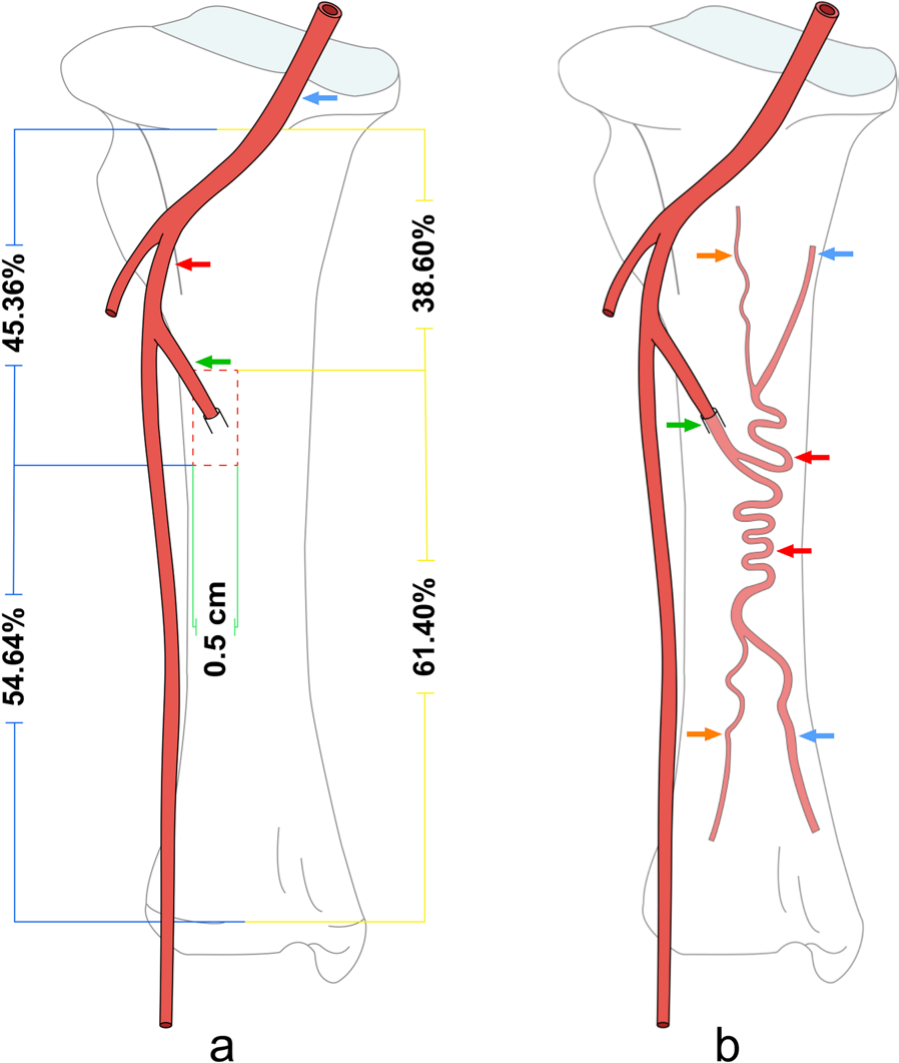
Morphology of internal and external tibial arteries. **a** The red area is the distribution range of the nutrient foramen, the blue arrow is the popliteal artery, the red arrow is the posterior tibial artery, and the green arrow is the nutrient artery. **b** The green arrow is the nutrient foramen, the red arrow is the medullary artery trunk, the blue arrow is the big branch, and the yellow arrow is the small branch

The shape of the nutrient artery and the location of the nutrient hole are shown in Table 1. A total of 10 nutrient arteries were found in 10 tibial specimens, which originated from the posterior tibial artery. The nutrient artery originates from the posterior edge of the posterior tibial artery, proceeds downward at the lower edge of the popliteus to between the flexor digitorum longus and the posterior tibial muscle, then enters the nutrient foramen at the outer edge of the tibia. The angle between the nutrient artery and the main tibial trunk differed substantially, ranging from 24.15° to 85.79°. The nutrient foramen was mainly distributed in the 0.5-cm range of the outer edge of the tibia. By measuring the distance between the nutrient foramen and the superior and inferior epiphyseal plate of the tibia, it was determined that the position of the nutrient foramen was approximately 4/10 of the tibia height (Fig. 6a).

**Table 1:** Angle of nutrient artery and location of the nutrient foramen (°, cm, *n* = 10)

Tibia label	Angle of nutrient artery	Actual position of nutrient foramen		Relative position of nutrient foramen
		Superior distance	Inferior distance	
01	68.79	5.85	9.31	38.60%
02	33.60	5.58	8.30	40.21%
03	75.52	7.12	9.03	44.07%
04	85.79	6.42	9.27	40.92%
05	57.17	6.48	9.15	41.45%
06	63.29	6.30	9.07	40.99%
07	28.51	6.63	7.98	45.36%
08	37.11	5.64	8.18	40.80%
09	51.80	6.13	9.61	38.95%
10	24.15	5.67	8.97	38.74%
Max	85.79			45.36%
Min	24.15			38.60%
x ± s	52.57 ± 21.12			41.01% ± 2.22%

Superior distance, distance between the nutrient foramen and the epiphyseal plate above the tibia; Inferior distance, distance between the nutrient foramen and the epiphyseal plate below the tibia; Relative position of nutrient foramen, Superior distance/(Superior distance + Inferior distance); x ± s, mean ± the standard deviation

The length and diameter of the trunk of the nutrient artery and the diameter of the branches of the posterior tibial artery are shown in Table 2. Before entering the bone the branches of the nutrient artery were short and thick, and their diameter of 8 arteries was larger than that of the posterior tibial artery. The maximum diameter of the nutrient artery is 1.80 mm, and the minimum diameter is 1.00 mm. After entering the bone the diameter of the nutrient artery is basically unchanged, extending inward and downward, and the average diameter of part of the intraosseous trunk is 0.79 mm.

**Table 2:** Lengths and diameters of arteries (mm, *n* = 10)

Parameters	Maximum	Minimum	Mean ± SD
Trunk length of the nutrient artery	6.90	3.20	5.22 ± 1.34
Diameter of the initial segment of the nutrient artery	1.80	1.00	1.29 ± 0.21
Diameter of the osseous segment of the nutrient artery	1.14	0.60	0.79 ± 0.18
Diameter of the inferior segment branches of the posterior tibial artery	1.80	0.52	1.07 ± 0.39

The distribution of nutrient arteries in bone exhibits regularity. The nutrient artery of the tibia obliquely penetrates the bone cortex through the nutrient foramen, and forms an acute angle with the long axis of the tibia. In the nutrient tube the artery has no branches. After passing through the bone cortex and entering the medullary cavity, the nutrient artery exhibits S-shaped winding in the medullary cavity, then divides into the ascending and descending medullary artery trunks. The upper and lower trunks of the medullary artery each send out 2–3 branches, and there are anastomoses between the branches. There is a large gap in the caliber of the branches, and often there is one big one and two small ones, or one big one and one small one. The small branch leads to both ends of the tibia and nourishes the epiphyseal plate and the nearby cancellous bone, and the big branch is mainly responsible for the blood supply of the tubular bone cortex, sometimes anastomosing with the branches of the periosteal artery (Fig. 6b).

## Discussion

### Selection of experimental materials

The purpose of this study was to explore a method for making cast specimens that could facilitate direct observation of the intraosseous artery of the tibia. Human tibia samples are relatively difficult to obtain, so it is more feasible to use animal tibia specimens instead. The selection factors of animal tibia specimens include tibia length, shape, vascular distribution, and caliber size. Throop et al. [17] reported that deer tibias are closer to human tibias in terms of length and morphology than tibias of other animals such as pigs and sheep, and mechanical test results obtained using them are also similar to those obtained using human tibias. They suggested that deer tibia should be used as a standard model for orthopedic implants. However, the report does not mention whether there is any difference in the distribution of intraosseous and extraosseous blood vessels between deer tibia and human tibia, and there is no similar literature for reference. Pigs are often replaced by goats and other animals in in vivo experiments because of their rapid growth and aggressiveness. However, there is high similarity between pig bone and human bone in terms of bone anatomy, shape, and composition [18]. Kotsougiani et al. [19] dissected the hindlimbs of 8 pigs and collected data on the source, length, and caliber of nutrient arteries in their tibias. The results they reported were highly similar to those derived from human tibias dissected by Anetai et al. [9]. Therefore, using porcine tibias as an experimental material to investigate the distribution of intraosseous arteries can help researchers to deepen their understanding of human intraosseous arteries.

### Blood supply to the tibia and treatment of fracture

After studying the anatomy of the lower limbs of fresh cadavers, Nelson and Kelly [20, 21] proposed that the blood supply to the tibia can be divided into three parts; the epiphyseal artery, nutrient artery, and periosteal artery. After entering the medullary cavity, the nutrient artery is divided into ascending and descending medullary artery trunks. The branches of the medullary artery near the inner surface of the bone cortex enter into the bone cortex [22]. Part of the capillary network radiating from the ascending and descending branches can pass through the bone cortex, and anastomose with the capillaries of the periosteal artery [23]. In the current study the structure of ascending and descending medullary arteries in pig tibia was similar to that in humans. The peripheral branches entered the bone cortex, and some of them penetrated the bone cortex and anastomosed with the periosteal artery to form a circular structure. The nutrient artery plays an important role in the blood supply of the tibial shaft. Levack et al. [24] performed quantitative magnetic resonance imaging (MRI) of 8 fresh frozen lower limbs and confirmed that the bone cortical area of the tibial shaft was dominated by intraosseous blood supply. If the intraosseous blood vessels are injured, the probability of nonunion after fracture is increased. At present, the commonly used clinical tibial fracture fixation instruments can easily cause damage to intraosseous blood vessels. According to a retrospective study of 105 patients with tibial fractures treated with external fixators by Almansour et al. [25], at least 38% of the patients had damaged tibial nutrient arteries, which may be conservative given that damage to the nutrient arteries itself cannot be detected by computed tomography. This view is also supported by Brinker et al. [26], who reported a similar situation in patients treated with tibial intramedullary nailing. After recognition of the importance of blood supply for fracture healing, existing instruments have been redesigned to avoid damaging intraosseous blood vessels during implantation; but in practice, considering factors such as operation time and firm fixation of the fracture, surgeons still tend to use methods they are familiar with in an effort to avoid intra-operative complications. There is now a consensus among most surgeons that retaining the residual periosteum as much as possible during the operation is desirable [27], but it is difficult to popularize and implement the concept of protecting intraosseous vessels.

### Significance of this study

The study of intraosseous vessels of the tibia has been challenging. Because of the hardness of the bone tissue, it is impossible to fully expose the blood vessels in the bone, as can be done via dissection in other organs. Although angiography, MRI, and other detection methods have verified the existence of intraosseous vessels, this is not sufficient for researchers who want to establish new surgical methods and instruments. They need a visual model that clearly shows the intraosseous vessels.

A method of vascular casting and exposure of the intraosseous artery of the tibia was established in the current study. The popliteal artery was separated and exposed, and after intubation, epoxy resin was injected into the popliteal artery, and the intraosseous vessels of the tibia were exposed by acid–base etching. The origin and 3D distribution of blood vessels in the tibia can be seen clearly. With the combination of 3D printing and transparent perfusion, this transparent tibia model has the advantages of easy implementation, low cost and short time-consuming. It can be used in human specimens to make a transparent human tibia model. Such a model will help surgeons and medical students to have a new understanding of the anatomy of the tibia and play a positive role in promoting medical education. After completing enough data collection and clarifying the distribution law of the intraosseous arteries, combined with CT, MRI and other examinations on this basis, it will be possible to identify the distribution and damage of the intraosseous arteries in a short period of time after the fracture occurs, reducing the risk of surgery and traditional Chinese medicine. The probability of intrinsic injury to the intraosseous artery also provides a theoretical basis for the development of new surgical procedures to restore blood supply to the fracture site.

Of course, the accuracy of the digital model of the 3D-scanned bone and intraosseous arteries remains to be verified, however this does not affect its value as a reference object for further research. The new tibial model containing the intraosseous artery will help to complement existing fracture analysis models and will help researchers develop new surgical modalities and instruments that avoid damage to the intraosseous blood vessels. The resulting data can be used in different materials and in different situations 3D printing is more cost-effective and repeatable.

### The future of intraosseous vessels of the tibia

At present the standard treatment of tibial fracture is to provide a blood supply to the fracture end indirectly, such as via autogenous cancellous bone transplantation, vascular pedicled bone flap transplantation, or periosteal transplantation [28]. There is no surgical method to directly restore blood supply to the fracture end after intraosseous vascular injury. This has a lot to do with our lack of a clear understanding of the distribution of blood vessels in the tibia. A skin flap is a tissue mass with its own blood supply that can survive independently. The earliest application of it in human history can be traced back to the sixth century BC. With the rapid development of super microsurgery in recent years, the clinical application of skin flap transplantation has expanded from plastic surgery to other types of surgery. The caliber of blood vessels that can be anastomosed is constantly being revised downwards. Xu et al. [29] successfully anastomosed blood vessels with a caliber of only 0.2 mm, proving that it is possible to restore blood supply via microsurgery. The current study successfully demonstrated the intraosseous vessels of pig tibia. This method can be used to make human transparent tibia specimens, which will have more research value. With the combination of a microsurgical technique and a skin flap transplantation technique it is possible to establish a new surgical method for one-stage treatment of tibial fracture, and restore a blood supply to the fracture end to prevent nonunion.

## Limitations

The present study had some limitations. There is currently no evidence that the distribution of intraosseous arteries in pig tibia is similar to that in humans, so the results of the study cannot be directly applied in the clinic. If human tibia specimens can be obtained for study, the results will be more meaningful. Another limitation relates to the fact that the complete intraosseous blood circulation system of the tibia includes arteries and veins. There was no vein perfusion or casting in the present study, though we plan to do this in future experiments. Lastly, the sample size was small, and the anatomical characteristics of the intraosseous artery of pig tibia were not analyzed and summarized in detail. In follow-up experiments, we will increase the sample size.

## Conclusion

In this study, a method for making transparent specimens of porcine tibia showing intraosseous arteries was successfully developed. The establishment of the cast model of the intraosseous artery provides methodological guidance for further study of the anatomical characteristics of the intraosseous artery and the blood supply distribution of the tibia. The preparation of transparent tibial specimens will help researchers to establish new models to develop new surgical methods and instruments, and facilitate more informative medical education, so that more surgeons have a more intuitive understanding of the concept of intraosseous vessels.

## Data Availability

All the data and materials are available from the corresponding author on request.

## Abbreviations

3D: Three-dimensional
CT: Computed tomography
MRI: Magnetic resonance imaging
